# Supplementary material for: ‘Seeing’ proximal representations: Testing attitudes to the relationship between vision and images
Source: PLoS One. 2021 Aug 20;16(8):e0256658. doi: 10.1371/journal.pone.0256658 (PMC8378678; doi:10.1371/journal.pone.0256658)
Supplement: Attachment — Submitted filename: Reply To Reviewers R2.docx [file pone.0256658.s003.docx]

PONE-D-21-09371
Can we see our proximal representations?
PLOS ONE

We thank the Editor and Reviewers once more for their consideration of our manuscript. Please find below our reply to the one comment requiring action.

Reviewer 1:

In their reply the authors note that "the experiment is not a study of size constancy", and they also say "we were investigating inclination/propensity rather than ability"

Ok, that is an important point. But in the abstract I read "we investigated whether adults can treat their own vision as if it were an uncorrected 2D image". The word CAN in particular implies that this is a study of the ABILITY to access 2D information despite size constancy. So some more changes to the words is necessary, other readers will read this and conclude that this is a study of size constancy.

Reply:

We agree that the use of 'can' is too ambiguous. We have changed this line in the abstract to read as follows:

In the present experiment we investigated how willing adults are to examine their own vision as if it were an uncorrected 2D image, much like a photograph.
